# Recent Advances in Mitochondria-Targeted Gene Delivery

**DOI:** 10.3390/molecules23092316

**Published:** 2018-09-11

**Authors:** Yoon-ha Jang, Kwang-il Lim

**Affiliations:** 1 Department of Chemical and Biological Engineering, Sookmyung Women’s University, Seoul 04310, Korea; miin_yj@sookmyung.ac.kr; 2 Institute of Advanced Materials and Systems, Sookmyung Women’s University, Seoul 04310, Korea; 3 Research Institute of ICT Convergence, Sookmyung Women’s University, Seoul 04310, Korea

**Keywords:** mitochondrial gene delivery, mitochondrial disease, mitochondrial genomes, heteroplasmy, mitochondrial matrix

## Abstract

Mitochondria are the energy-producing organelles of cells. Mitochondrial dysfunctions link to various syndromes and diseases including myoclonic epilepsy and ragged-red fiber disease (MERRF), Leigh syndrome (LS), and Leber hereditary optic neuropathy (LHON). Primary mitochondrial diseases often result from mutations of mitochondrial genomes and nuclear genes that encode the mitochondrial components. However, complete intracellular correction of the mutated genetic parts relevant to mitochondrial structures and functions is technically challenging. Instead, there have been diverse attempts to provide corrected genetic materials with cells. In this review, we discuss recent novel physical, chemical and biological strategies, and methods to introduce genetic cargos into mitochondria of eukaryotic cells. Effective mitochondria-targeting gene delivery systems can reverse multiple mitochondrial disorders by enabling cells to produce functional mitochondrial components.

## 1. Introduction

**Figure 1:**
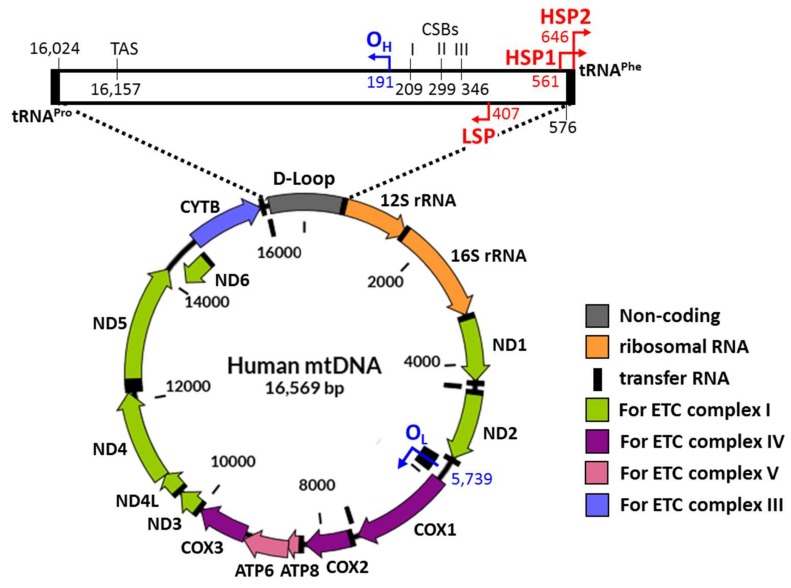
Schematic diagram of human mitochondrial genomic DNA. Mitochondrial DNA is 16,569 bp long. mtDNA encodes 13 mRNAs required to produce components for the electron transport chain complex and 2 rRNAs and 22 tRNAs for mitochondrial translation. D-Loop is the major non-coding region that contains the promoter components called HSP1, HSP2, and LSP. The replication origins of heavy and light strands known as O_H_ and O_L_ control the mtDNA replications. Compared with O_H_, locating within the D-Loop, O_L_ is located between the region encoding three tRNAs and the region encoding two other tRNAs. The names of the protein-coding genes are shown in the diagram. LSP = light strand promoter. HSP1 and HSP2 = heavy strand promoters. CSB = conserved sequence block. TAS = termination-associated sequence.

Mitochondria are intracellular organelles that produce adenosine triphosphate (ATP), which is a usable form of energy for cells. In eukaryotes, cells convert nutrients to ATP through oxidative phosphorylation that is carried out by the electron transport chain complex in the membranes of mitochondria. In addition, mitochondria take part in the regulation of cell growth and synthesis of multiple biological molecules such as pyrimidine, heme, neurotransmitters, and hormones. Mitochondrial proteins play key roles in these cellular processes and they are encoded by mitochondrial DNA (mtDNA) or nuclear DNA (nDNA). In human cells, mitochondria are present in a wide range of numbers from a few hundred to about one thousand depending on their tissue origins and one mitochondrion contains two to eight copies of mtDNA in the mitochondrial matrix [1,2,3]. mtDNA encodes 13 mRNAs that are used to produce multiple protein components for the electron transport chain complex (Figure 1). nDNA encodes genes for not only the remaining components of the complex but also proteins involved in various mitochondrial functions.

Mutations in mtDNA or parts of nDNA alter the sequences of mitochondrial proteins, which ultimately causes mitochondrial dysfunction. Neurodegeneration, seizures, myopathy, cardiomyopathy, deafness, optic atrophy, and developmental delays are common symptoms of mitochondrial disorders. Multiple therapeutic strategies to treat mitochondrial dysfunctions that were caused by mtDNA mutations, in particular, have been based on the consideration of mtDNA heteroplasmy. Mitochondria normally contain more than one type of genome including possibly the wild type mtDNA and mutant variants in some cases. Therefore, a small percentage of mtDNA with mutations around multiple copies of wild type mtDNA is not sufficient to lead to a disease phenotype. However, if the percentage is over the threshold (typically >80%), mitochondrial disease occurs [4]. Based on this heteroplasmic feature of mtDNA, it might be possible to recover mitochondrial functions by supplementing the impaired genes above the clinical threshold with the corresponding normal genes in gene therapy.

Development of gene delivery systems that can pass through the mitochondrial double membranes is a key to the success of mitochondrial gene therapy. Mitochondria are roughly structured by two phospholipid bilayers including the mitochondrial outer membrane and the inner membrane, which are separated by the intermembrane space and enclose the mitochondrial matrix. Small molecules such as ions, ATP, and proteins smaller than 10 kDa can freely diffuse across the channels of the outer membrane. The mitochondrial inner membrane, however, forms a barrier that selectively transports molecules into the mitochondrial matrix. The mitochondrial inner membrane is composed of cardiolipin, which has four alkyl tails, which render the membrane impermeable to hydrophilic molecules. Outflow or inflow of molecules related to mitochondrial functions is only enabled with the help of various proteins such as transporters and translocases.

**Figure 2:**
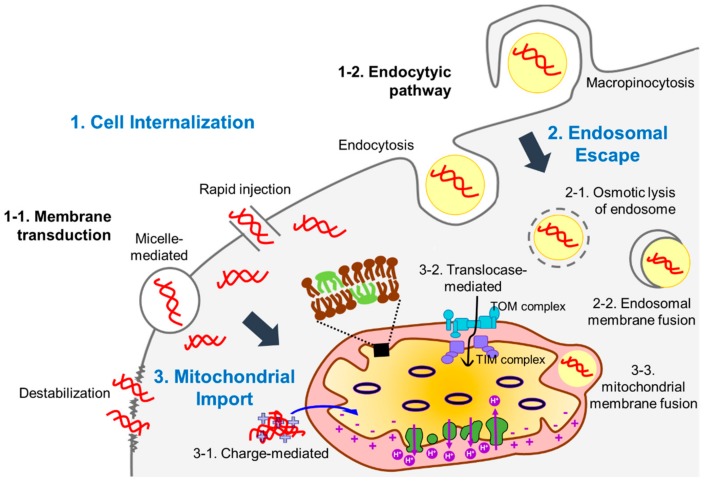
Barriers against delivery of exogenous DNA into mitochondrial matrix. There are three main barriers (words in blue) that need to be overcome to deliver DNA into mitochondria: the cell membrane (against cell internalization, (1), the endosomes (against endosome escape, (2), and mitochondrial double membranes (against mitochondrial import, (3). The hydrophilic feature of DNA molecules links to their low cell membrane permeability. Various strategies have been applied to enhance the passage of DNA molecules through the cell membrane. Physical, chemical, and biological methods enhance the DNA molecule internalization into cells by generating pores in the plasma membrane (via membrane transduction) or depending on the cellular endocytic pathways. In the latter case, cargo DNA needs to escape from endosomes before being degraded by hydrolytic enzymes in the late endosomes or lysosomes. Multiple carrier molecules can facilitate endosomal escape of DNA molecules often via osmotic lysis of endosomes. Liposome-based carriers have fusogenic activity with an endosomal membrane to enhance the release of DNA molecules from endosomes to the cytoplasm. DNA or carrier molecule-DNA complex in the cytoplasm needs to pass through mitochondrial double membranes to reach the mitochondrial matrix, which is the final destination. Commonly, cationic molecules are used as carrier molecules to facilitate the transfer of DNA molecules into the matrix because the matrix is negatively charged by the actions of an electron transport chain complex. In addition, mitochondrial targeting signal peptides that can be recognized by receptors of translocases in the mitochondrial membranes are often linked to carrier molecules. Like fusion with endosomal membranes, liposome-based carrier molecules can be imported into the mitochondrial matrix through membrane fusion when their lipid composition is properly designed.

Delivery of DNA molecules encoding the corrected versions of genes into mitochondria is a promising strategy to treat mitochondrial disease. Due to the impermeability of the mitochondrial inner membrane to hydrophilic molecules, however, passage of DNA through the mitochondrial double membrane remains a challenge. In this review, we summarize various recent physical, chemical, and biological approaches to transfer DNA into mitochondria and discuss the advantages and limitations for these approaches (Table 1 and Figure 2). Methods for enhancing the expression of the cargo DNA are also introduced. At last, we highlight the applications of these strategies to treat mitochondrial diseases.

## 2. Physical Approaches

Physical gene delivery systems can provide a simple and direct way to transfer exogenous genes into cells because they penetrate the cell membrane without the need for carrier molecules to enhance accessibility to the cells or intracellular organelles. One such physical method known as microinjection uses a fine glass micropipette or needle to directly introduce liquids containing genetic materials into cells. Another example known as particle bombardment uses helium gas flow at a high velocity to propel DNA-coated micropellets into target cells. In addition, pulse electric fields and ultrasonic sound waves have been used in electroporation and sonoporation, respectively, to penetrate the cell membrane. Recently, these physical approaches have been applied for exogenous gene delivery specifically into the mitochondria.

Using hydrodynamic limb vein injection, Yasuzaki et al. demonstrated that naked plasmid DNA could be delivered into rat liver mitochondria in vivo [5]. Hydrodynamic injection uses hydrodynamic pressure generated by injecting a large volume of fluid into a blood vessel to permeabilize the capillary endothelium and induce pores in the plasma membranes of surrounding cells [29]. They injected a solution containing plasmid DNA into the distal vein of limbs in anesthetized rats [5,6]. As a result, they verified mitochondrial delivery of the plasmid DNA by quantifying it in the mitochondria-enriched fraction based on quantitative polymerase chain reaction (PCR).

Biolistic technology, which is another physical method, introduces DNA into the plasma membrane of target cells by coating it onto heavy metal particles and moving these complexes at high speed. Gene delivery to mitochondria using biolistic technology was carried out in *Saccharomyces cerevisiae* [8]. Since mitochondrial genetic systems of single-cell eukaryotes are well known, methods to introduce DNA into mitochondria in single-cell eukaryotes can provide a useful reference for multi-cell eukaryote systems. First, DNA was precipitated onto tungsten particles (<1 µm), which were then penetrated into the plasma membrane by high-pressure helium gas-driven acceleration. After bombardment, the DNA was randomly imported into mitochondria and cells with transformed mitochondria were subsequently selected. Biolistic gene delivery is advantageous in that it can be used regardless of the cell type and non-toxic metal particles are available. However, to date, there have been few reports of its use for mitochondrial gene delivery in mammalian cells.

In contrast to chemical and biological methods, physical methods do not require carrier molecules (Table 1). Therefore, they do not introduce any toxicity associated with these molecules. However, physically imported DNA is evenly distributed throughout the cytoplasm and it enters the mitochondrial matrix randomly, which makes it difficult to deliver DNA specifically to the mitochondria. Another limitation of physical methods is that the target cells can be damaged in the process of penetrating the cell membrane (Table 1).

## 3. Chemical Approaches

Most methods developed for mitochondrial gene delivery are chemical-based. While physical methods enhance the efficiency of gene delivery by increasing cellular uptake of DNA with physical forces, chemical methods are often based on specific chemical interactions with mitochondria. Mitochondrial membranes have hydrophobic characteristics and are also negatively charged. Therefore, it is necessary to enclose negatively charged DNA with carrier molecules that have cationic and amphiphilic properties for efficient delivery. In chemical strategies, carrier molecules encounter several challenges before reaching the mitochondria (Figure 2). In most cases, they must first cross the cellular membrane via endocytosis or translocation. If carrier molecules follow the endocytic pathway, they are not accessible to mitochondria until they escape from the endosome. Accordingly, it is important to find molecules that facilitate the passage of these carriers from the endosome. In addition, there should be strategies to direct the floating DNA-carrier complex in the cytoplasm to mitochondria. Thus, mitochondria-targeting molecules are conjugated with DNA-binding motifs or presented on the surface of DNA-enclosing vesicles composed of dendrimers, surfactants, or liposomes. Many carrier molecules can be easily improved by altering the incorporated mitochondria-targeting motifs.

Cardoso et al. used cationic surfactants with two hydrophilic head groups and two hydrophobic groups as DNA carriers for mitochondrial delivery [9,30]. Even at a low concentration, the cationic gemini surfactants could form micelle-like structures surrounding DNA strands with a negative charge in aqueous solution. This occurred by hydrophobic and electrostatic attractive interactions between the surfactant molecules and DNA [31]. Based on the interactions of gemini-surfactant-DNA complexes with vesicles mimicking the mitochondrial membrane structure, they hypothesized that four modified gemini surfactant-DNA complexes would differentially cross the cellular membrane through either endolysosomal routes or membrane translocation. The former releases plasmid DNA into the mitochondrial surroundings and the latter directly interacts with the mitochondrial membrane. Although they did not identify the precise mechanism underlying the delivery, confirmation of expression of the gene transferred to the mitochondria demonstrated successful entry of the carrier plasmid into the mitochondria [30]. Gemini surfactants usually deliver exogenous DNA into mitochondria passively in that the DNA is delivered to the mitochondrial surroundings instead of the interior. However, specificity for mitochondria can be improved through chemical modifications. Moreover, gemini surfactant-mediated gene delivery can reduce potential cytotoxicity because only small amounts of chemicals are required to transfer DNA.

To improve targeting to mitochondria, rhodamine 123, which is a biocompatible dye with a delocalized positive charge and green fluorescence, was incorporated into nanoparticles carrying plasmid DNA [11]. The lipophilicity combined with the delocalized positive charge of this dye specifically allows nanoparticles to pass through both the plasma and mitochondrial membranes and to ultimately accumulate in the mitochondrial matrix, which has a negative charge. The rhodamine-plasmid DNA nanoparticle complex can be easily prepared by co-precipitation with calcium carbonate. An addition of cellulose increased the stability of the complex in aqueous solution and reduced its size to facilitate penetration of the plasma and mitochondrial membranes [11]. In a subsequent study, successful delivery of plasmid DNA into mitochondria using a rhodamine-based carrier was confirmed by fluorescence imaging of the rhodamine [10].

Liposome-based vesicles can be used to encapsulate plasmid DNA and deliver the complex into mitochondria across the plasma membrane and cytoplasm. For example, the dequalinium (1,1′-(1,10-decamethylene-*bis*-[aminoquinaldinium])-chloride) vesicle (DQAsome) is an amphiphilic and cationic lipid-based vesicle made by self-assembly of dequalinium [12]. The intrinsic ability of dequalinium to accumulate in the mitochondrial matrix was confirmed using a cardiolipin-rich liposome, which is a model of the mitochondrial membrane [14]. When the DQAsome/DNA complex (DQAplex) came into contact with the cardiolipin-rich liposome, cargo DNA was released. In a subsequent study with a mammalian cell system, it was demonstrated that DQAplexes could be released from the endosome and subsequent dissociation of the DQAplex occurred selectively at the mitochondria [12]. Although DQAsomes have high specificity for mitochondria, their use has several limitations such as low transfection efficiency, cytotoxicity, and difficulty in generating homogenous DQAsome solutions. To overcome these limitations, Bae et al. devised the DQA80, which incorporated lipid molecules 1,2-dioleoyl-3-trimethylammonium-propane (DOTAP) and 1,2-dioleoyl-*sn*-glycerol-3-phosphoethanolamine (DOPE) to improve transfection efficiency and increase endosomal escape, respectively [15]. Mixtures of dequalinium and DOTAP/DOPE in different proportions were assessed for their ability to form a carrier for mitochondrial gene delivery. Complexes of plasmid DNA and DQA80s with 10% of DOTAP and DOPE showed enhanced stability and effective mitochondrial targeting.

Using such functionalization methods, existing carriers for mitochondrial gene delivery have been greatly improved. Harashima’s group developed the MITO-Porter, which is a liposome-based nanocarrier that targets mitochondria, and they have continuously improved the MITO-Porter in several studies [16,17,18,19]. The major advantage of the MITO-Porter is that it can be easily multi-functionalized by adding ligands. To enhance cellular uptake, which is the first step in delivery of exogenous DNA into mitochondria, the MITO-Porter surface was modified with octa-arginine (R8) peptides mimicking the transactivator of the transcription (TAT) peptide. The TAT peptide fuses to proteins and is commonly used for the delivery of biological molecules crossing the cell membranes [17,18,32,33]. The cationic property of TAT peptide facilitates interactions with negatively charged phospholipid and carbohydrate components of the cell membrane [32].

R8-modified liposomes were found to have improved fusion with mitochondrial membranes and could introduce nucleic acids, peptides, proteins, sugars, or chemicals into mitochondria [16]. MITO-Porter surface-modified with R8 was constructed by multi-layering methods including encapsulation of nanoparticles with a mitochondria-fusogenic inner envelope and endosome-fusogenic outer envelope [18]. R8 peptides in the outer layer allowed efficient internalization of carriers and those in the inner layer fused with the mitochondrial membrane [18]. Lastly, the MITO-Porter, which has an inner envelope composed of sphingomyelin, was found to have both a high mitochondrial membrane fusogenic activity and low cytotoxicity [17].

Another attempt was made to enhance the mitochondrial targeting activity of MITO-Porter by functionalizing it with cationic amphipathic cell-penetrating peptides called KALA-peptides, which are composed of repeated lysine-leucine-alanine residues instead of R8 peptides [19,20]. The resultant KALA-MITO-Porter showed comparable cellular uptake with Lipofectamine 2000, which is a well-known transfection reagent, and improved cellular uptake when compared to the R8-MITO-Porter. Moreover, expression of cargo DNA delivered by the KALA-MITO-Porter was the highest among the three carriers. However, in the BCA protein assay of cellular protein concentration in MITO-Porter-treated cells, KALA-MITO-Porter-treated cells showed lower levels of proteins when compared to R8-MITO-Porter-treated cells, which indicates higher cytotoxicity. It was hypothesized that this cytotoxicity was caused by the destabilization of the mitochondrial membrane by the KALA peptide.

A new approach to constructing carrier systems for mitochondrial gene delivery is the conjugation of the triphenylphosphonium (TPP) cation to liposome-containing DNA as a ligand for mitochondrial targeting [21]. TPP is a lipophilic cation with a delocalized positive charge, which facilitates accumulation into the mitochondrial matrix due to the membrane potential across the mitochondrial inner membrane [23]. Boddapati et al. incorporated TPP into the lipid bilayer of liposomes by conjugating a stearyl residue to form stearyl triphenyl phosphonium (STPP), which is an amphiphilic molecule. As a result, the liposome surface-modified with STPP easily targeted mitochondria and successfully delivered cargo molecules. However, cytotoxic effects of STPP-modified liposomes were observed and, accordingly, the stearyl moiety was substituted with a biocompatible polyethylene glycol (PEG)-phosphatidyl ethanolamine (PE) polymer to incorporate TPP in the liposomes [22]. Novel surface-modified liposomes with TPP-PEG-PE delivered cargo into mitochondria without cytotoxicity.

Biswas et al. conjugated TPP to the surface of polyamidoamine (PAMAM) dendrimers, which can encapsulate biomolecules in their core due to the multi-valency effect of dendrimer-facilitated conjugation of many ligands [23]. Dendrimers are classified by the generation number, which refers to the size and number of repeated functional groups, and generation 5 has been used specifically as a carrier in gene or drug delivery applications. Due to their size, generation 5 PAMAM dendrimers can solubilize multiple hydrophobic entities and simultaneously diffuse through the tissue [34,35,36]. Generation 5 PAMAM dendrimers also have a high positive surface charge, which means they can condense negatively charged DNA by electrostatic interaction and subsequently form PAMAM-DNA complexes [23,37]. The resultant complex has a net positive charge that allows efficient endosomal escape. Subsequently, TPP-conjugated PAMAM dendrimers were localized to mitochondria with reduced cytotoxicity, which was often caused by nonspecific targeting. The latter was achieved by conjugating an acetyl group, which neutralizes the highly cationic dendrimer and allows a high level of association with the anionic cell membrane [23].

The chemical approaches for mitochondrial gene delivery discussed here require chemically synthesized carrier molecules to load DNA, pass through intracellular barriers, and ultimately release DNA into the mitochondria (Table 1 and Figure 2). Their common advantage is that the carrier molecules can be easily and widely modified to overcome limitations such as low efficiency of mitochondrial targeting or high cytotoxicity to target cells. Studies of carrier molecules have focused on their ability to bypass intracellular barriers and pass through the mitochondrial double membrane mostly via membrane fusion. These characteristics can be improved by linking ligands that target specific mitochondrial molecules such as receptors rather than a broad feature of the membrane. Since the ultimate goal of exogenous DNA delivery into the mitochondrial matrix is to supplement the genes that produce defective mitochondrial proteins, it is necessary to confirm whether transferred DNA is properly released and transgene expression is possible.

## 4. Biological Approaches

Mitochondrial proteins encoded by nDNA account for more than 99% of total mitochondrial proteins [38]. Mitochondrial proteins expressed in the cytosol as mitochondrial precursor proteins enter the mitochondria via mitochondrial targeting signal peptide (MTS)-mediated translocation. Most MTSs are located at the amino-terminus of precursor proteins and are cleaved by proteolysis upon import into mitochondria. The MTS presequences form an amphipathic α-helix that has hydrophobic residues on one side and positively charged residues on the other side. MTS-conjugated proteins enter the mitochondrial matrix through the translocase of the mitochondrial outer membrane (TOM) and that of the mitochondrial inner membrane (TIM). The MTS at the *N*-terminus of a precursor protein is preferentially recognized by the TOM20-TOM22 receptor subcomplex, which contains clusters of negatively charged residues. The protein passes through the import pore formed by TOM40 [39,40]. Consequently, precursor proteins enter the channel formed by TIM17 and TIM23 across the inner membrane in the presence of a membrane potential. The *N*-terminal domain of TIM23 encodes an MTS receptor that has negatively charged residues, which interact with the positively charged amphipathic MTS. Through this interaction, MTS conjugates can pass through the mitochondrial double membranes. Accordingly, conjugation of MTS to carrier molecules that have a DNA-binding function has been a strategy to enable specific mitochondrial gene delivery. For successful MTS-mediated gene delivery, the selection of an effective MTS is required. However, highly hydrophobic proteins and macromolecules are not suitable for MTS-mediated gene delivery [41].

In an initial study, a peptide nucleic acid (PNA) was used as a DNA carrier molecule conjugated with MTS [35]. PNA, which is a DNA oligomer with a polyamide bond instead of a deoxyribose phosphate backbone, was conjugated to an MTS peptide to retain base-specific hybridization [24,42]. PNA and MTS-PNA were biotinylated to evaluate intracellular distribution and introduced into different cell lines including human fibroblasts, HeLa cells, HepG2 cells, and SY5Y cells. Treated cells were subsequently labeled with streptavidin-fluorescein. While PNA without MTS was localized to the nucleus, MTS-conjugated PNA was imported into the mitochondria to some degree. The level of mitochondrial targeting was low because nuclear localization tendency of PNA counteracted mitochondrial targeting. As expected, MTS-mediated PNA entered mitochondria through the TOM and TIM complexes [25]. Transferred oligonucleotides can selectively inhibit the replication of mutated mtDNA if the oligonucleotide is designed to be the anti-sense of mutated mtDNA. Even though this approach cannot transfer large genes, it is a promising early-stage application of MTS in mitochondrial gene delivery.

Similarly, Chuah et al. linked a nonspecific DNA-binding peptide to an MTS to form a mitochondrial gene carrier [26]. A KH peptide comprising alternating lysine and histidine residues that are positively charged was designed to bind to DNA that has the negatively charged phosphate backbone. This peptide enabled cargo DNA to easily pass through the cellular membrane by condensing the DNA. Histidines in the KH peptide are known to facilitate endosomal escape by the effect of a proton sponge, which increases osmolality of the endosome and causes swelling due to accumulation of histidines in the endosome [43]. They conjugated the pre-sequences of yeast cytochrome c oxidase subunit IV and human hepatic enzyme ornithine transcarbamylase to the KH peptide and tested their applicability as mitochondrial gene carriers. As a result, MTS-KH peptides and naked plasmid DNA were electrostatically self-assembled and the resultant complex successfully entered mitochondria, which was followed by the expression of the exogenous plasmid DNA [26].

Viruses have been extensively used in gene therapy as vehicles due to their innate ability to insert their genome into the host cell. Among the various viral vectors used for gene delivery to the nucleus, the adeno-associated virus (AAV) vector has also been used for gene delivery to mitochondria [27]. AAV has an ssDNA genome consisting of the *rep* gene encoding non-structural proteins (REP78, REP68, REP52, and REP40) and the *cap* gene encoding capsid proteins (VP1, VP2, and VP3). The two genes are flanked by inverted terminal repeat (ITR) sequences, which provide *cis*-acting sequences required for replication, packaging, and integration of the viral genome. For application in gene therapy, the promoter and transgene are inserted between the two ITR sequences in the viral vector and both the *rep* and *cap* genes are provided in trans for packaging the vector particles [44].

With respect to the AAV entry process, capsid proteins participate in internalization into the host cell, intracellular transport, and localization. To redirect AAV to mitochondria, Yu et al. constructed MTS-AAV by inserting the cytochrome oxidase subunit VIII (COX8) pre-sequence into VP2, which is one of the capsid proteins in AAV serotype 2 [27]. Among the three capsid genes, it was feasible to insert signal peptides into VP2 because, even though both VP1 and VP2 are involved in nuclear translocation, VP1 affects viral infectivity [45]. In addition, the *N*-terminus of VP2 can tolerate insertion of large peptides [46]. In Leber’s hereditary optic neuropathy (LHON), the NADH ubiquinone oxidoreductase subunit 4 (ND4) gene is defective. The wild type human or rodent mitochondrial genes were loaded into the MTS-AAV genome for delivery to the mitochondria of human cells with ND4 mutations or the cells of LHON rodent models, respectively. Since the ND4 gene encodes for a subunit of electron transport chain complex I, LHON cells lack ATP. When the MTS-AAV-mediated ND4 gene was introduced into LHON disease cell lines, rescue of ATP synthesis was observed. In LHON rodents, visual loss and optic atrophy as well as phenotypic symptoms of LHON were also ameliorated by MTS-AAV-mediated ND4 gene therapy. The exact entry mechanism of the MTS-AAV system was not identified. However, in a subsequent study, the research group demonstrated through next generation sequencing that the transferred genes remained episomal [47]. The major advantage of the MTS-AAV-mediated gene delivery system is that it can be applied in vitro and eventually in vivo once clinical safety is established. Additionally, it can deliver hydrophobic proteins and macromolecules, which is a limitation of MTS-peptide conjugates [28]. However, the size of the cargo gene that can be delivered by AAV is still limited. Thus, the use of retroviral vectors or lentiviral vectors that can accommodate larger sizes of genes may be required.

## 5. Combinatorial Approaches

We have classified the various methods of gene delivery to mitochondria into physical, chemical, and biological ones (Table 1). Since each strategy has advantages and disadvantages, individual methods can be combined to improve the import of genes into mitochondria. We discuss several examples where methods have been successfully combined.

When DNA was hydrodynamically injected into mouse liver, condensed DNA rather than naked DNA was used [6]. Plasmid DNA was biologically condensed with protamine, which is a positively charged protein molecule. During injection, the condensed plasmid DNA was internalized into cells by using hydrodynamic force. It was hypothesized that condensed plasmid DNA has a small aggregated form with a positive charge, which enhances mitochondrial association [6]. This strategy helps to overcome the limitation of non-selective hydrodynamic delivery of naked DNA into mitochondria.

Similarly, rhodamine 123, as described above, formed nanoparticles with plasmid DNA by co-precipitation [11]. Incorporation of polymers such as cellulose and gelatin, which are derived from biological components, into nanoparticles condensed the DNA-nanoparticle complex since cellulose and gelatin bind Ca^2+^, which reduces electrostatic repulsion between hydroxyl and carboxyl groups in the complex [48]. Additionally, cellulose and gelatin have properties such as biocompatibility and stability in aqueous solution, which enhanced the transfection efficiency of the rhodamine 123-based mitochondrial gene delivery system. 

Yamada et al. linked MTS to MITO-Porter to integrate the mitochondria-specific targeting activity of MTS with the MITO-Porter delivery system [49]. MTS was conjugated with DOPE (MTS-DOPE) using 4-(*N*-maleimidomethyl)-cyclohexane-1-carboxylic acid *N*-hydroxysuccinimide ester (SMCC) as a bifunctional cross-linker for displaying MTS on the surface of liposomes. MTS-DOPE was added to a lipid solution for preparing liposomes. The resultant MTS-modified MITO-Porter enhanced mitochondrial selectivity and, thereby, more frequently fused with the mitochondrial membrane as compared with non-modified liposomes. These findings suggest the possibility that MTS-modified MITO-Porter could be an efficient carrier for gene delivery through mitochondrial membrane fusion [49].

Unlike existing approaches based on the properties of mitochondrial structure, a novel strategy based on the biological function of mitochondria was suggested. A study showed that CdSe/ZnS core/shell quantum dot (QD) nanoparticles coated with glutathione (GSH) were selectively accumulated in the mitochondrial matrix despite the negative charge of GSH [50]. GSH is a key in maintaining the appropriate mitochondrial redox environment. It is synthesized in the cytosol and easily passes through the mitochondrial outer membrane through porin channels. The anionic property of GSH is unfavorable for entry against the electrochemical gradient present in the mitochondrial inner membrane. However, GSH transporters anchored to the mitochondrial inner membrane including the dicarboxylate carrier (DCc) and the 2-oxoglutarate carrier (OGc) make the entry possible [51]. Zheng et al. observed that GSH-QD clusters were localized to mitochondria by TEM imaging and organelle staining. The specific mitochondrial targeting mechanism of GSH was not identified, but it was shown that GSH can act as a surface ligand for nanocarriers by targeting mitochondria [50].

## 6. Cargo DNAs

The main objective in developing exogenous gene delivery systems targeted to mitochondria is to compensate for defective proteins caused by mtDNA mutations. Therefore, successful mitochondrial gene delivery requires not only introduction of exogenous DNA into the mitochondrial matrix but also expression of functional proteins from introduced DNA. Lyrawati et al. designed and generated an artificial mini-mitochondrial genome that contained regulatory domains present in the endogenous mitochondrial genome and a reporter gene to demonstrate transgene expression in mitochondria [13].

The mitochondrial genome is made of circular and double-stranded DNA that is composed of the heavy (H) strand and the light (L) strand (Figure 1). Most of the mitochondrial genome comprises coding regions [52]. These consist of the L-strand replication origin (O_L_) and the displacement loop (D-loop) (Figure 1) [53]. The D-loop includes many regulatory domains such as the major H-strand promoter (HSP2), the L-strand promoter (LSP), the H strand replication origin (O_H_), the conserved sequence blocks (CSBs), and the termination-associated sequence (TAS) [54,55]. These non-coding regions play roles in replication, transcription, and translation of mtDNA. Both ends of the D-loop are flanked by genes encoding tRNA^Phe^ on the H-strand and genes encoding tRNA^Pro^ on the L-strand [56,57]. These regions are known to be a major site for the control of gene expression (Figure 1).

The newly constructed mini-mitochondrial genome possessed its own genetic expression system similar to that of the mitochondrial genome [13]. For stable expression of transgenes in mitochondria, the mini-mitochondrial genome was constructed to have a D-loop region with flanking sequences that encoded tRNA^Phe^ and tRNA^Pro^ and an O_L_ region with flanking sequences that encoded tRNA^Asn^, tRNA^Cys^, and tRNA^Tyr^. The transgene encoding the reporter green fluorescent protein (GFP) was designed to be located between the D-loop region and the O_L_ region. To ensure the reporter gene was expressed in mitochondria, the codon for tryptophan (TGG) in the interior of the gene was changed to the stop codon TGA, which is read as tryptophan in vertebrate mitochondria [56]. Accordingly, translation in the cytosol of the resultant reporter gene with the modified genetic code (mtGFP) was terminated prematurely and could not produce green fluorescence. Translation only in the mitochondria can produce functional GFP with full length. The resultant mini-mitochondrial genome was delivered into the mitochondria of the RAW 264.7 macrophage cell line via DQAsome-mediated gene delivery.

After transfection, a small fraction of transfected cells showed intense green fluorescence within the cytoplasm but not in the nucleus. The fraction of cells producing the modified reporter protein encoded by mtGFP was increased over 14 days post-transfection and the presence of the mini-mitochondrial genome in mitochondria was confirmed by PCR 30 days after transfection. The sustained expression and stability of the mini-mitochondrial genome as an artificial mitochondrial genome are advantageous features for mitochondrial therapeutics [13].

Based on the artificial mini-mitochondrial genome, researchers have constructed cargo DNA molecules with promoters originally encoded in the mitochondrial genome and reporter genes or mitochondrial endogenous genes optimized to the mitochondrial codon system [7,27]. HSP2, which drives transcription of 12 of the 13 protein-coding genes in mitochondria, has been used to mediate transgene expression [53,58] (Figure 1). Yamada et al. showed for the first time that the cytomegalovirus (CMV) promoter, which has been widely used to achieve a high level of nuclear transgene expression in mammalian cells, could trigger the transcription of mitochondrial genes efficiently [19,59]. An HSP-driven reporter gene, which consists of a luciferase gene adjusted to the mitochondrial codon system (mtLuc), produced no luminescence when the corresponding DNA construct was delivered to HeLa cells by Lipofectamine 2000. The novel pCMV-mtLuc vector was constructed by substitution of HSP with the CMV promoter and resulted in a high level of luciferase activity under the same delivery approach. Moreover, the luciferase activity of transferred CMV-mtLuc was similar before and after treatment with α-amanitin, which is a specific inhibitor of RNA polymerase II. This result implies the transcription of CMV-mtLuc in mitochondria. These findings indicate the presence of new mechanisms involved in the process of transcription in mitochondria in addition to the well-known mitochondrial transcriptional system. This strategy may allow the production of functional proteins required for normal mitochondrial activity in mitochondrial disease when various approaches are applied for mitochondrial gene delivery.

In contrast to the above-mentioned approaches that aimed to provide cells with corrected versions of mitochondrial genetic materials, several research groups applied new approaches to eliminate mitochondrial genomes with mutations. They targeted disease-causing point mutations within mitochondrial genomes including T8993G that links to neuropathy ataxia, retinitis pigmentosa (NARP) syndrome, and Leigh syndrome, A8344G associated with MERRF, and A3243G, which may lead to mitochondrial encephalopathy, lactic acidosis, and stroke-like episodes (MELAS) [60,61,62,63,64]. To specifically destroy mitochondrial genomes carrying the targeted mutations, endonucleases such as *Sma* I were first employed. When the gene that encoded the MTS-tagged *Sma* I was introduced into cells, the fraction of mitochondrial genomes with the mutation of T8993G was greatly decreased via cutting of the mutated sequence by the action of the endonuclease [63]. Given a set of existing endonucleases with defined recognition sites, target DNA sequences are limited. To more easily target other mitochondrial DNA mutations, zinc fingers and transcription activator-like effectors (TALEs) have been combined to DNA nucleases [60,61,62,64]. These two types of DNA-binding proteins can be engineered to have specificities for new DNA sequences of interest. For the same goal of easy targeting various mitochondrial mutations, clustered regularly interspaced short palindromic repeats (CRISPR)/Cas9 proteins can be also introduced into cells through the addition of the corresponding encoding genes. If the supply of corrected genetic materials and elimination of mutated mitochondrial genomes are applied at the same time, more effective treatment of mitochondrial malfunctions might be possible.

## 7. Applications as Disease Therapies

Genetic mutations in mtDNA cause a number of diseases and the major sites of mutations are well known [65]. For example, mitochondrial diseases such as MERRF disease are caused by the A8344G mutation in the tRNA^Phe^-coding gene. Neurogenic muscle weakness, ataxia, retinitis pigmentosa (NARP), and Leigh syndrome are caused by the T8993G mutation in the ATP6 gene. Other genetic mutations have been linked to Kearns-Sayre syndrome, Ataxia neuropathy syndrome, and Alpers-Huttenlocher [66].

LHON is a well-studied mitochondrial disease. It involves retinal ganglion cell death and degeneration of optic nerves. Over 90% of LHON patients carry one of three point mutations in their mtDNA: G3460A in the ND1 gene, G11778A in the ND4 gene, and T14484C in the ND6 gene. Among these mutations, G11778A accounts for more than 70% of the cases of LHON [67,68]. Yu et al. successfully achieved delivery of the wild type ND4 gene and ND4 expression using MTS-AAV in an LHON cell line harboring G11778A [27]. The treated cells showed restoration of ATP synthesis, which is deficient in the LHON cell line. Additionally, the MTS-AAV-mediated ND4 gene delivery was demonstrated to be effective in in vivo testing. In a mouse model of LHON, mutant mice injected with MTS-AAV carrying the wild type ND4 gene showed improved visual function after one year post injection when compared with control groups injected with either AAV not flanked by MTS or MTS-AAV carrying only GFP gene. Histological cross-sections of the optic nerves confirmed that the mutant mice injected with MTS-AAV carrying the wild type ND4 gene had thicker optic nerves, which indicated that optic atrophy was relieved by the wild type ND4 allele [27].

MELAS are other well-known symptoms of the mitochondrial disease caused by various point mutations in the NADH dehydrogenase gene or mitochondrial tRNA-coding genes [69]. Gene delivery using MITO-Porter functionalized with KALA peptides (KALA-MITO-Porter) was conducted with cells obtained from a patient suffering from MELAS-like symptoms including epilepsy, hearing loss, and an elevated lactate level. KALA-MITO-Porter-mediated delivery of the luciferase gene and wild type ND4 gene under the CMV promoter were tested in fibroblasts harboring the G625A mutation in the mitochondrial tRNA^Phe^-coding gene. In this study, the biocompatibility and efficiency of both the KALA-MITO-Porter-mediated gene delivery system and the CMV-driven ND4 expression were investigated. As a result, cell viability was not affected by KALA-MITO-Porter regardless of cargo DNA and cell type, but there was no detectable luciferase activity in diseased cells. To facilitate binding of KALA-MITO-Porter to diseased cells and improve cellular uptake and mitochondrial targeting, a mitochondrial RNA aptamer was added. Using this approach, they achieved efficient mitochondrial transgene expression. However, a more practical approach would have been to include the delivery of cargo DNA that was deficient in diseased cells. In this case, it includes the gene encoding tRNA^Phe^. Nevertheless, the results of this research highlight the importance of practical application of mitochondrial gene delivery systems.

Until now, there have been few attempts to directly deliver genes into the mitochondria of cells or animals that are actually under mitochondrial dysfunction. Instead, several groups have attempted to transfer genes to cells under mitochondrial disease states without mitochondria-targeting. Previously, the yeast NDI1 gene encoding NADH-quinone oxidoreductase was reported as being capable of modulating oxidative phosphorylation in 293 human kidney cells. In another study, this gene restored the respiratory NADH oxidase activity of complex I-deficient mammalian cells [70,71]. Based on these findings, the *S. cerevisiae* NDI1 gene was packaged into recombinant AAV (rAAV-NDI1) and was introduced into human C4T cells, which carry a homoplasmic frameshift mutation in the ND4 gene. The C4T cells cannot form the NADH dehydrogenase complex and, thus, do not grow in galactose medium because they lack glycolytic activity. The C4T cells transduced with rAAV-NDI1 produced NDI1 proteins in the cytosol, which were then imported into the mitochondria due to the NADH-binding site facing the mitochondrial matrix. As a result, the C4T cells highly expressing NDI1 could grow in a galactose medium [70]. To demonstrate if this mitochondrial gene delivery system is applicable to diseased cells or animals, construction of both various diseased models and efficient delivery systems is required.

## 8. Concluding Remarks

In contrast to chromosomes in the nucleus, mitochondria possess many copies of their genome and, thus, only a small percentage of mutated alleles do not cause mitochondrial disease. This feature can be exploited to treat mitochondrial diseases caused by genetic mutations in mtDNA by complementing essential genes. The major challenge of gene delivery is passage through the mitochondrial double membranes to transfer cargo DNA into the mitochondrial matrix (Figure 2). Based on the structure of mitochondria, cationic amphiphiles linked to DNA binding or encapsulating motifs have been used as carriers for transgene delivery. Another challenge is the stable expression of the transgene to produce functional proteins to restore mitochondrial activity. To date, few studies have reported successful delivery of genetic cargo and transgene expression in cells or animals with mitochondrial disease. Various approaches have been used to deliver wild type genes into the mitochondrial matrix and have been improved steadily to overcome the challenges including passage through the mitochondrial membranes, low and unstable transgene expression, or cytotoxicity derived from carrier molecules (Table 1). Most studies reviewed here provide targeting strategies to transfer genetic cargo into mitochondria with ongoing improvements. However, compared to nuclear gene delivery methods, mitochondrial gene delivery methods are currently far from being clinically applicable. We think that new approaches and trials would be required to effectively deliver parts of or the entire wild-type mitochondrial genome to cells of interest in order to achieve long-term expression of transgenes. Additionally, more disease models need to be developed to cover the wide range of genetic mutations in mitochondria. 

## Figures and Tables

**Table 1:** Summary of approaches for mitochondrial gene delivery.

Classification	Key Acting Component	Delivery-Target Systems	Strategy	Advantages	Limitations	Ref.
Physical	Hydrodynamic injection	Rat, Mice	Cell penetration by hydrodynamic force	Simplicity	No mitochondria-targeting	[5,6,7]
	Biolistics	Yeast	Cell penetration by bombardment	Cell type-independent	Potential cell damage, No mitochondria-targeting	[8]
Chemical	Gemini surfactants	HeLa cell	Formation of cationic micelle-like structure with DNA	Works in small dose	Weak specificity to mitochondria	[9]
	Rhodamine 123	Normal human dermal fibroblast (NHDF) adult donor cell	Precipitation of DNA using lipophilic molecule with delocalized positive charge	Can be traced due to fluorescence	Expression from transferred DNA has not been confirmed	[10,11]
	DQAsomes	BT 20 cell	Transport of DNA by ampiphilic and cationic lipid-based vesicle	High specificity to mitochondria	Low transfection efficiency, Cytotoxicity	[12,13,14]
	DQA80s	Primary human dermal fibroblasts, HeLa cell	Lipid incorporation into DQAsomes	Improved transfection efficiency	Delivery of DNA around mitochondria not into the matrix	[15]
	MITO-Porter	HeLa cell, Rat	Membrane fusion by lipid-based nano carrier	Easy surface modification		[16,17,18,19]
	R8-MITO-Porter	Rat	Enhancing cellular uptake by functionalization with cationic peptide	High fusogenic activity with mitochondrial outer membrane	Low fusogenic activity with mitochondrial inner membrane, Moderate cytotoxicity	[16,17]
	KALA-MITO-Porter	HeLa cell, Mice	Enhancing cellular uptake by functionalization with membrane destabilizing peptide	Improved transfection efficiency	High cytotoxicity	[19,20]
	STPP-liposome	4T1 cell, Mice	Conjugation of stearyl residue to lipophilic and cationic TPP for ampiphilic property	Selective accumulation in mitochondria	Cytotoxicity	[21]
	TPP-PEG-PE liposome	HeLa, 4T1 cell, Mice	Substitution of stearyl moiety with biocompatible PEG-PE polymer	Decreased cytotoxicity	Transgene expression not confirmed	[22]
	TPP-PAMAM dendrimer	HeLa, MCF-7, 4T1, NIH 3T3 cell	DNA condensation by high positive surface charge, endosomal escape by free tertiary amine groups	Efficient endosomal escape and high serum resistance	Transgene expression not confirmed	[23]
Biological	MTS-PNA	Myoblasts, Fibroblasts, NT 2, IMR 32, HeLa, HepG2, C2C12 cell	MTS-guided localization of DNA hybridized with PNA to mitochondria	High specificity to mitochondria via actions of translocase	Only can transfer short nucleic acids	[24,25]
	MTS-KH peptide	HEK 293 cell	Mitochondrial localization of MTS-conjugated DNA-binding peptide and exogenous DNA complex	Can transfer large DNA with high specificity to mitochondria		[26]
	MTS-AAV	Neuronal G11778A NT 2 cybrid, HEK 293T cell, Mice	Mitochondrial localization of DNA by inserting MTS into the AAV capsid	Proven effects of transgene expression	Inability to carry large DNA	[27,28]
